# Preliminary evaluation of autologous pericardium ring for tricuspid Annuloplasty: a two-year follow-up study

**DOI:** 10.1186/s13019-019-1017-5

**Published:** 2019-11-12

**Authors:** Wei Jiang, Xiao-Mao Long, Si-Cong Li, Yong-Long Zhong, Bang-Fu He, Hui Lin

**Affiliations:** Department of Thoracic Cardiovascular Surgery, The People’s Hospital of Guangxi Zhuang Autonomous Region, No.6 Taoyuan Road, Qingxiu District, Nanning, 530021 Guangxi China

**Keywords:** Autologous pericardium tissue ring, Annuloplasty, Tricuspid regurgitation (TR), Edwards-MC3 annuloplasty, DeVega annuloplasty

## Abstract

**Objective:**

To evaluate the effectiveness of autologous pericardium ring in tricuspid annuloplasty surgery for the treatment of tricuspid regurgitation (TR).

**Methods:**

From December 2010 to December 2012, a total of 107 patients with secondary TR underwent tricuspid annuloplasty. The patients were divided into three groups: autologous pericardium ring group (*n* = 38), Edwards-MC3 ring group (*n* = 35), and DeVega group (*n* = 34). The patients were followed-up for two years. The survival rates and free from hospital readmission rates were measured and analyzed. The patients also received transthoracic echocardiography (TTE) in order to obtain TR regurgitant jet area to right atrial area (S_TR_/S_TA_), diastolic tricuspid annuloplasty diameter (DTAD), right atrial diameter (RAD), and right ventricular diameter (RVD).

**Results:**

One patient from DeVega group and one patient from autologous pericardium ring died from low cardiac output syndrome during the perioperative period. In the two-year follow-up period, each group has one instance of death for unclear reasons. One month after operation, the S_TR_/S_TA_, DTAD, RAD, and RVD values in all groups were significantly lower than the pre-operation values (*P* < 0.05). During the two year follow-up period, DTAD values of patients from DeVega group increased significantly as compared to the values at one month post operation (P<0.05), which is different from the other two groups in which DTAD values remained stable (P>0.05). In both pericardium ring group and Edwards-MC3 group, S_TR_/S_RA,_ remained stable (P>0.05) during the follow-up period, whereas S_TR_/S_RA_ of the DeVega group had showed a tendency of increase (although statistically insignificant, P>0.05). There was no significant difference in the survival rates among three study groups (*P* > 0.05), but the rate of free from hospital readmission in the DeVega group was significantly lower than those in the other two groups (*P* < 0.05) during the two-year follow-up period.

**Conclusions:**

Autologous pericardium tissue based ring annuloplasty demonstrated remarkable clinical utility for treating tricuspid regurgitation. It shows similar beneficial results to Edwards-MC3 annuloplasty within a short-term follow-up period, and outperforms the widely used DeVega annuloplasty. Autologous pericardium tissue annuloplasty represents a promising technique for tricuspid annuloplasty and holds great potential for treating tricuspid valve dysfunctions.

## Introduction

Functional tricuspid insufficiency is one of the most common heart valve diseases [1, 2]. It is closely related to pulmonary hypertension and right ventricular dilation, [3, 4] which are usually secondary to left side valve dysfunctions [5]. It has been demonstrated that the tricuspid leaflets dilation is a key pathological factor that contributes to the tricuspid insufficiency [6]. In clinics, treatment or interference of tricuspid insufficiency such as tricuspid regurgitation (TR) becomes an inevitable procedure following left side heart surgery because the progression of tricuspid insufficiency increases the chance of post-operative mortality and ultimately defeats the purpose of corrective cardiac operation [4, 7]. Therefore, a safe and effective tricuspid valve annuloplasty (TVA) is a critical to the effective treatment of heart valve disease [8]. Currently, there are two major techniques for TVA, namely, DeVega’s suture annuloplasty and prosthetic ring annuloplasty [9–11]. However, DeVega’s technique is plagued by certain drawbacks such as the possibility of broken stitches, fallen-apart or contracted valve leaflets. The recurrence rate of tricuspid insufficiency in DeVega patients can reach 10 to 40% in the middle to long term [9, 12]. Therefore, there is an increasing preference for prosthetic ring annuloplasty in tricuspid valve surgery. With the prosthetic ring annuloplasty technique, the recurrence rate of TR is about 8 to15% at early stage after the operation [13]. Nevertheless, certain issues remained unresolved. For example, the implantation of prosthetic mechanical ring affects the contraction and dilation of the ventricle, which sometimes causes the rupture of the ring, thrombosis, and endocarditis [14]. Furthermore, the relative high cost of prosthetic ring is a chief deterrent to its widespread application in clinical cardio operations in China. In comparison, autologous pericardium tissue has been widely used in cardiac operations due to its easy accessibility, low immunogenicity, and excellent durability [15–17]. To the best of our knowledge, there is no reporting on the application of autologous pericardium tissue in tricuspid annuloplasty in a clinical setup. Herein we describe the application of autologous pericardium ring in tricuspid annuloplasty and present our evaluation of this new ring annuloplasty technique by comparing it with two well-known procedures, i.e., Edwards-MC3 annuloplasty [18] and DeVega annuloplasty [19] in a two-year follow-up study.

## Subjects and methods

Inclusion criteria of the patients: (1) moderate or severe grade of TR; (2) sinus rhythm; (3) atrial fibrillation (AF) prior to operation and was converted to sinus rhythm when received atrial fibrillation ablation during the course of the operation.

Exclusion criteria of the patents: (1) past history of tricuspid annuloplasty surgery; (2) the TR regurgitant jet area to right atrial area (S_TR_/S_TA_) is less than 20%; or (3) informed consent was not signed by the patient.

From December 2010 to December 2012, 107 patients who are diagnosed with tricuspid regurgitation are recruited for our study. Based on the operations they were going to receive, the patients were randomly divided into three groups: (1) autologous pericardium ring (APR) group (*n* = 38); (2) Edwards-MC3 group (*n* = 35); and (3) DeVega group (*n* = 34). The details of the clinical data of the patients are summarized in Table 1. The clinical data of the patients among three study groups are statistically insignificant.

**Table 1:** Clinical data of the patients in three study groups

Parameter	APR(n = 38)	Edwards-MC3(*n* = 35)	DeVega(*n* = 34)
Age (years)	42.56 ± 11.23	41.28 ± 11.13	43.45 ± 12.18
Male/Female	18/20	17/18	17/17
Surface area (m^2^)	1.58 ± 0.17	1.59 ± 0.18	1.60 ± 0.16
NYHA functional classification			
II (%)	17 (45%)	16 (46%)	14 (41%)
III (%)	21 (55%)	19 (54%)	20 (59%)
Sinus rhythm before operation (%)	9 (24%)	8 (23%)	8 (24%)
Atrial fibrillation (AF) to sinus rhythm during operation (%)	29 (76%)	27 (77%)	26 (76%)
Heart valve dysfunction			
Mitral valve (%)	20 (53%)	21 (60%)	18 (53%)
Aortic valve (%)	0	0	0
Mitral valve and aortic valve (%)	18 (47%)	14 (40%)	16 (47%)
Degree of TR			
Mild (%)	23 (61%)	22 (63%)	21 (62%)
Severe (%)	15 (39%)	13 (37%)	13 (38%)

Note: Doppler ultrasound was performed to assess the grading of severity of TR. Based on the effective regurgitant orifice area (EORA)/diameter of the right ventricle, < 20% is graded as mild; 20 to 40% is graded as moderate; > 40% is graded as severe

**Fig. 1:**
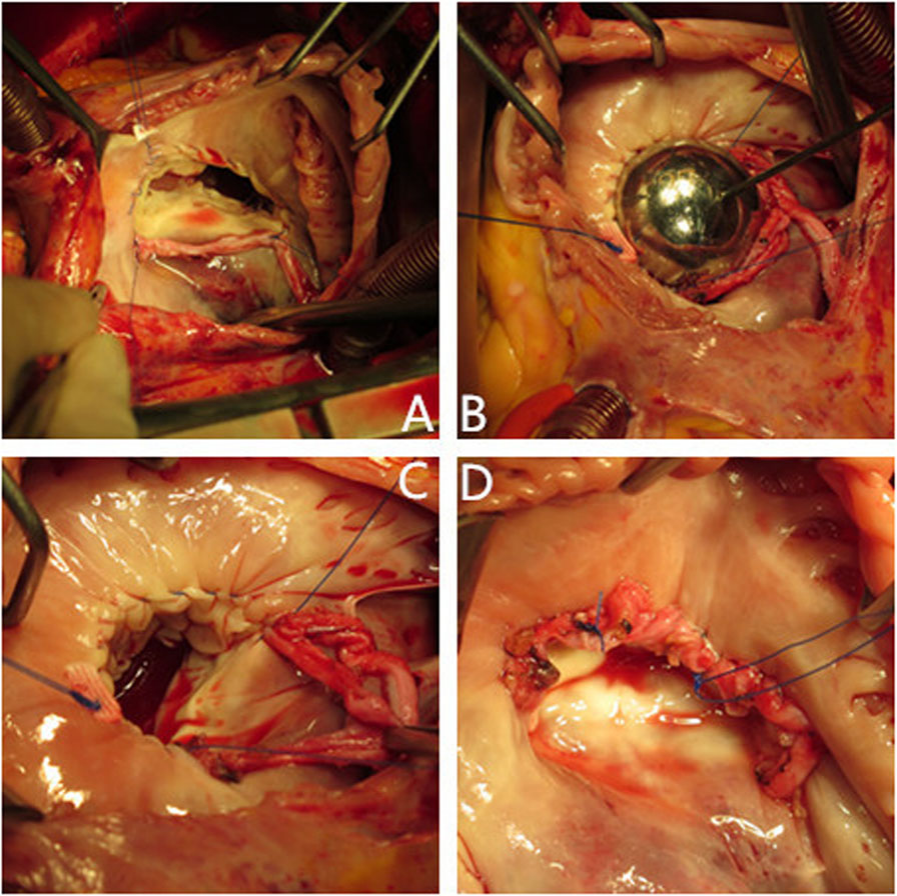
**a**-**d** Autologous pericardium ring annuloplasty

Prior to performing the tricuspid annuloplasty procedures, the patients underwent the following treatments: A sternotomy was conducted under anesthesia. Subsequently, radiodefrequency ablation procedures were performed with cardiopulmonary bypass and non-stopping of the heart function. After completion of the ablation, the heart was stopped to allow for aortic or mitral valve replacement or repairing. The heart is then allowed to resume beating followed by one of the tree tricuspid annuloplasty procedures described below.

DeVega suture tricuspid annuloplasty [20]: A 3–0 polypropylene suture with Teflon felt was placed in the annulus, beginning at the commissure of coronary sinus and septal leaflet. The suture was continued along the tricuspid annulus counterclockwise, and ended at the anterior leaflet to form a purse-string. The second arm of the suture was used to make a similar purse string, but in an alternative sequence to bring the stitches in and out of the annulus. The desired annulus size of the tricuspid was determined according to the values presented in Table 2. A valve sizer was used to measure the size of the annulus. After desirable annulus size was achieved, a knot was drawn.

**Table 2:** Reference annulus size of the tricuspid based on surface area

Number	Surface area (m^2^)	annulus size of the tricuspid (mm)
1	0.25	13.4
2	0.30	14.90
3	0.35	16.20
4	0.40	17.30
5	0.45	18.20
6	0.50	19.20
7	0.60	20.70
8	0.70	21.90
9	0.80	23.00
10	0.90	24.00
11	1.00	24.90
12	1.20	26.20
13	1.40	27.70
14	1.60	28.90
15	1.80	29.10
16	2.00	30.00

Edwards-MC3 ring tricuspid annuloplasty: based on the measurement of length of the septal annulus and/or the area of the anterior leaflet by a valve sizer, [21] prosthetic rings with appropriate size were selected. The suture was performed using a 6 × 14 double armed needle starting from the commissure of anterior and posterior leaflets and continued through the commissure of coronary sinus and septal leaflet to form a “U” shape. An average of 9–11 stitches is needed. The prosthetic rings were secured by sewing the felt to the rings and tying up to form a knot. In total, 20 counts of size 28 rings, 10 counts of size 30 rings, and 5 counts of size 32 rings were used.

Autologous pericardium ring annuloplasty: the first suture started at the commissure of coronary sinus and septal leaflet, and continued counterclockwise through the commissure of anterior and posterior leaflets. The second suture started at the commissure of anterior and septal and continued clockwise to commissure of anterior and posterior leaflets (Fig. 1a). The two felts are tied up to form a knot. The size of the tricuspid annulus was adjusted according to Table 2 (Fig. 1b and c). Subsequently, fresh autologous pericardium tissue was sewed to the tricuspid leaflets (Fig. 1d).

### Follow-up study

The follow-up study was conducted at a one month interval for a period of two years following the operation. Based on the NYHA functional classification, patients with class IV designation were re-admitted for diuretics therapy. Color Doppler echocardiography was performed at the time of (1) prior to operation; (2) one month after operation; and (3) two years after operation to measure TR regurgitant jet area to right atrial area (S_TR_/S_TA_), diastolic tricuspid annuloplasty diameter (DTAD), right atrial diameter (RAD), and right ventricular diameter (RVD).

### Statistical analysis

The data was analyzed using SPSS17.0 software package and the results are represented with $\overline{X}$ ±s. Comparison among three study groups was carried out using t test and Pearson’s chi-squared test. Survival rate and hospital readmission rate were analyzed by *Kaplan-Meie*r method.

## Results

Perioperative period: During this period, none of the patients have shown paravalvular leakage complication after replacement with prosthetic valves in the Edwards-MC3 group. DeVega group and autologous pericardium ring group each have one instance of death caused by low cardiac output syndrome.

One month post-operation: Echocardiography was performed at this time point. In DeVega group, thirteen patients had recurrence of mild TR, two patients had recurrence of moderate TR, and one patient had recurrence of severe TR. The rest of the patients showed no sign of TR. In Edwards-MC3 group, eleven patients had recurrence of mild TR, three patients had recurrence of moderate TR, and one patient had recurrence of severe TR. The rest of the patients showed no sign of TR. In autologous pericardium ring group, ten patients had recurrence of mild TR and one patient had recurrence of moderate TR. The rest of the patients showed no sign of TR. Both DTAD and S_TR_/S_TA_ values are statistically insignificant among three groups (*P* > 0.05). All three groups showed significant decrease of S_TR_/S_TA_, DTAD, RAD, and RVD after the operation (*P* < 0.05).

Two-year follow-up period:

1. Survival rate.

**Fig. 2:**
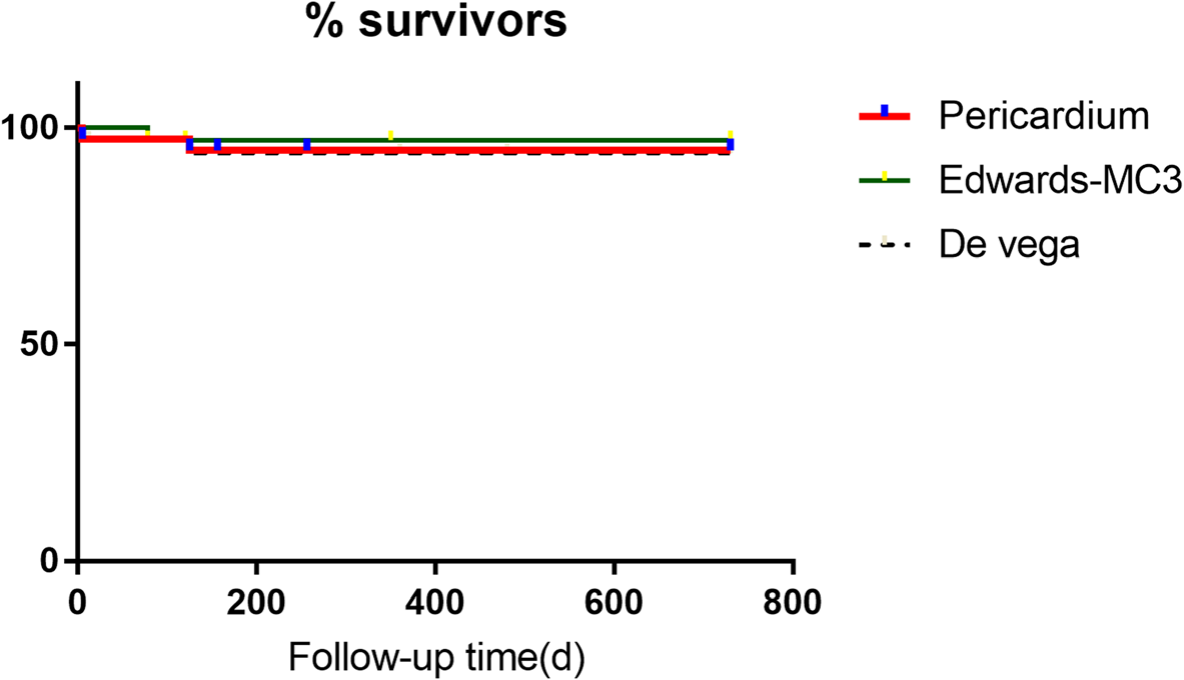
Survival rate in follow-up study

During the two-year follow-up study, two patients lost contact with us in each of the three study groups and therefore are excluded from our study. There was also one instance of death in each of the three study groups for unclear reasons. The survival rates across three study groups are statistically insignificant (Fig. 2).

2. Rate of free from hospital readmission.

**Fig. 3:**
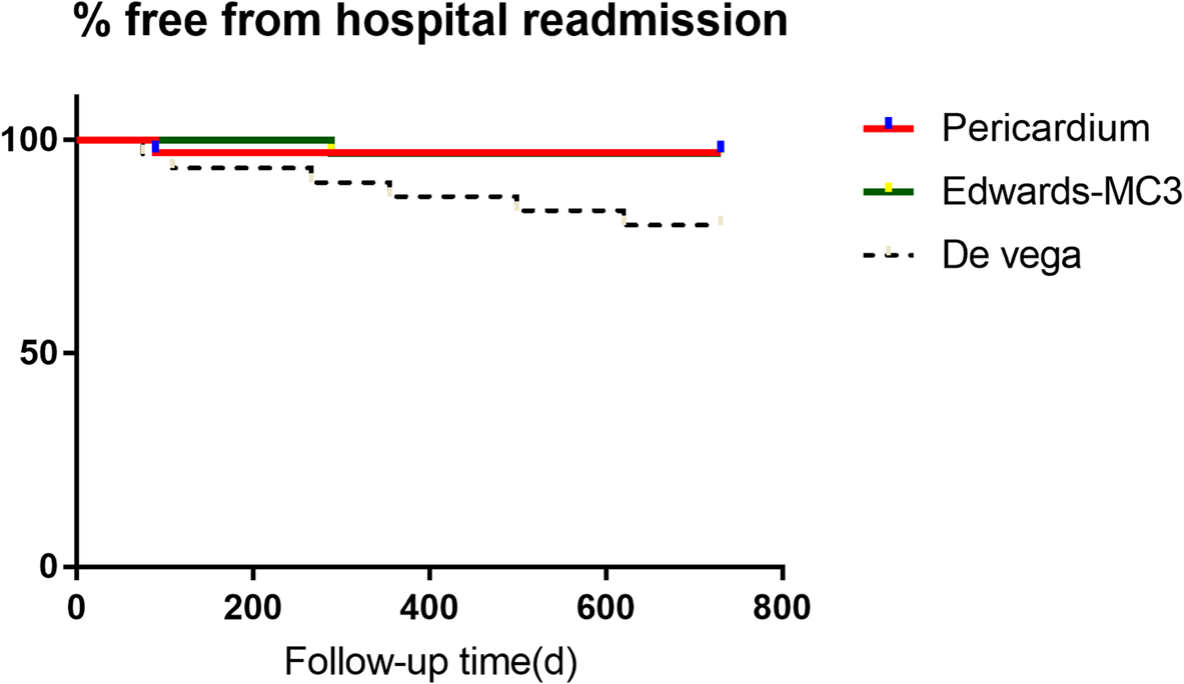
Rate of free from hospital readmission

For the patients remained in the study, none of them has shown any sign of paravalvular leakage or thrombosis complication after replacement with prosthetic valves in the Edwards-MC3 gruop. However, in DeVega group, seven patients were re-admitted for NYHA class IV heart failure, and the free from hospital readmission rate is 80% ± 12.8% at the end of two-year period. In Edwards-MC3 group, one patent was re-admitted for class IV heart failure, and the free from hospital readmission rate is 96.9% ± 15.3% at the end of two-year period, which is significantly higher than that of DeVega group, *P* = 0.0164). Similarly, the autologous pericardium ring group has one case of class IV heart failure and was re-admitted, and the survival rate is 97.1% ± 16.7% at the end of two-year period, which is the highest of three study groups and significantly higher than the DeVega group(*P* = 0.0203). The results of the hospital readmission rate are summarized in Fig. 3.

3. Transthoracic echocardiography.

Based on the transthoracic echocardiography data, we identified that in DeVega group, there were 16 cases of mild TR, 10 cases of moderate TR, and 2 cases of severe TR. In Edwards-MC3 group, there were 12 cases of mild TR, 3 cases of moderate TR, and 2 cases of severe TR. In the autologous pericardium ring group, there were 11 cases of mild TR and 1 case of moderate TR.

The DTAD values from the DeVega group significantly increased one month after operation (*P* < 0.05), and are also significantly higher than the other two groups at the same time point (P < 0.05). In comparison, the DTAD values for patients in both Edwards-MC3 and autologous pericardium ring groups remained stable (*P* > 0.05). Similarly, the S_TR_/S_TA_ values for patients in both Edwards-MC3 and autologous pericardium ring groups remained unchanged (P > 0.05). Although the increase of S_TR_/S_TA_ values for DeVega group patients are not statistically significant one month after operation, we observed an increasing trend of TR recurrence. The details of the results are summarized in Table 3.

**Table 3:** Comparison of the right heart functional parameters in three study groups

Parameters	APR group			Edwards-MC3 group			DeVega group		
	T = 0	T = 2 mo	T = 24 mo	T = 0	T = 2 mo	T = 24 mo	T = 0	T = 2 mo	T = 24 mo
S_TR_/S_TA_(%)	39 ± 12.22	8 ± 1.35	11 ± 2.42	37 ± 10.67	10 ± 2.48	12 ± 3.27	38 ± 12.12	12 ± 2.23	15 ± 4.16
DTAD (mm)	36 ± 7.25	27 ± 1.16	28 ± 2.18	35 ± 6.11	28 ± 2.15	29 ± 2.79	35 ± 6.27	27 ± 1.38	32 ± 3.26
RAD (mm)	47 ± 9.56	40 ± 7.69 ^a^	36 ± 5.38	46 ± 9.27	41 ± 7.33	36 ± 5.45	46 ± 9.56	42 ± 6.53	40 ± 5.38
RVD (mm)	50 ± 10.12	45 ± 8.45	40 ± 7.22	49 ± 10.28	46 ± 8.27	40 ± 7.48	49 ± 10.23	45 ± 8.76	43 ± 6.56

## Discussion

According to the pioneering work on the anatomical investigation of tricuspid insufficiency reported by Kay et al., [2] the dilation of the tricuspid annulus primarily occurs through the dilation of anterior leaflet. The septal leaflet usually has the least dilation due to space constraint. The degree of dilation normally comes in the order of 80 for anterior leaflet, 40% for posterior leaflet, and 10% for septal leaflet. Inspired by their findings, we envision that fresh autologous pericardium tissue ring can be utilized in tricuspid annuloplasty. The benefits of this design are threefold: (1) This approach not only allows for the restriction of anterior and posterior leaflets, but also secures the septal leaflet under the upper rim of the coronary sinus. (2) Autologous pericardium tissue can serve as a strong supporting frame, which enhances the stability of the tricuspid leaflets. Importantly, this stable ring framework addresses the issues that often affect the performance of suture annuloplasty such as broken suture and fallen-apart tissues. Furthermore, as endothelial cells migrate after the operation, the pericardium tissue is integrated into the tricuspid structure, which further prevents the dilation of the tricuspid annulus in the long term. (3) Since fresh autologous pericardium tissue exhibits high degree of compliance under stress, this approach preserves the proper contraction and relaxation of the right ventricle.

As illustrated in the results section, we observed significant decrease in S_TR_/S_TA_, DTAD, RAD, and RVD for all three study groups one month after operation. These results indicate all of these annuloplasty techniques are effective immediately after the operation. However, for DeVega group, the DTAD value increased significantly at the end of the follow-up study as compared to the Edwards-MC3 and autologous pericardium ring group. Thus, it is evident that both Edwards-MC3 and pericardium ring annuloplasty are better at stabilizing the tricuspid annulus and preventing its dilation. The distinction between DeVega group and Edwards-MC3 and pericardium ring group is further reflected in the recurrence rates of TR at 1 month and two years after the operation. Specifically, there was a trend of increasing TR observed in the DeVega group, albeit the increase is statistically insignificant. This is consistent with the observation reported by Katsi et al. where they concluded that the degree of TR is correlated with the dilation of tricuspid annulus diameter and the tricuspid annulus diameter is an independent factor to predict tricuspid insufficiency [22]. Nevertheless, the free from readmission rate for the DeVega group is lower than the other two groups and there are no other complications which would have originated from the prosthetic rings, suggesting that the recurrence of TR is the major risk factor for DeVega annuloplasty.

It is worthwhile to note that, the procedures for conducting autologous pericardium ring in tricuspid annuloplasty follow after left heart operations when the heart has resumed beating. Therefore, this approach reduces the time when the heart is stopped and ultimately reduces the risk for the operation.

## Conclusion

In summary, we developed a cost effective and operationally convenient method for tricuspid annuloplasty. Based on a two-year follow-up study, we observed this method offered comparable results to widely used Edwards-MC3 ring annuloplasty and holds a great potential in broader applications in treating heart valve diseases. Since this study is carried out in a two-year period, certain questions such as whether autologous pericardium needs to be fortified by glutaraldehyde remained to be explored. Moreover, a longer term such as a five-year follow-up study is well underway in our research group.

## Data Availability

The datasets generated and analyzed during the current study are available from the corresponding author on reasonable request.

## Abbreviations

DTAD: diastolic tricuspid annuloplasty diameter
RAD: right atrial diameter
RVD: right ventricular diameter
TR: tricuspid regurgitation
TTE: transthoracic echocardiography
TVA: tricuspid valve annuloplasty
